# The Initial Draining Lymph Node Primes the Bulk of the CD8 T Cell Response and Influences Memory T Cell Trafficking after a Systemic Viral Infection

**DOI:** 10.1371/journal.ppat.1003054

**Published:** 2012-12-06

**Authors:** Matthew R. Olson, Daniel S. McDermott, Steven M. Varga

**Affiliations:** 1 Department of Microbiology, University of Iowa, Iowa City, Iowa, United States of America; 2 Interdisciplinary Graduate Program in Immunology, University of Iowa, Iowa City, Iowa, United States of America; 3 Department of Pathology, University of Iowa, Iowa City, Iowa, United States of America; University of Pennsylvania, United States of America

## Abstract

Lymphocytic choriomeningitis virus (LCMV) causes a systemic infection in mice with virus replication occurring in both peripheral tissues and secondary lymphoid organs. Because of the rapid systemic dissemination of the virus, the secondary lymphoid organs responsible for the induction of the LCMV-specific CD8 T cell response are poorly defined. We show that the mediastinal lymph node (MedLN) serves as the primary draining lymph node following LCMV infection. In addition, we demonstrate that the MedLN is responsible for priming the majority of the virus-specific CD8 T cell response. Following resolution of the acute infection, the draining MedLN exhibits characteristics of a reactive lymph node including an increased presence of germinal center B cells and increased cellularity for up to 60 days post-infection. Furthermore, the reactive MedLN harbors an increased frequency of CD62L^−^ effector memory CD8 T cells as compared to the non-draining lymph nodes. The accumulation of LCMV-specific CD62L^−^ memory CD8 T cells in the MedLN is independent of residual antigen and is not a unique feature of the MedLN as footpad infection with LCMV leads to a similar increase of virus-specific CD62L^−^ effector memory CD8 T cells in the draining popliteal lymph node. Our results indicate that CD62L^−^ effector memory CD8 T cells are granted preferential access into the draining lymph nodes for an extended time following resolution of an infection.

## Author Summary

CD8 T cells are required for the elimination of infected host cells following an acute virus infection. In addition, memory CD8 T cells provide immunity to the host against a secondary infection. Much is known about the priming of CD8 T cells towards viruses that induce a localized infection, however the site responsible for priming the majority of CD8 T cells following a systemic viral infection remains unclear. Lymphocytic choriomeningitis virus (LCMV) induces an acute systemic viral infection when inoculated intraperitoneally, eliciting a robust CD8 T cell response. Although intraperitoneal LCMV infection results in rapid systemic viral replication, we demonstrate that the mediastinal lymph node (MedLN) serves as the initial draining lymph node and represents the primary site for the induction of the acute CD8 T cell response. In addition, we observe that CD62L^−^ effector memory CD8 T cells are preferentially recruited into the draining MedLN for up to 60 days following LCMV infection. Collectively, these studies indicate that the draining lymph node remains poised to defend the host against a secondary encounter with a pathogen for a prolonged time following the primary infection.

## Introduction

Lymph nodes (LN) play a critical role in initiating the adaptive immune response following viral infections. For example, intravenous (i.v.) vesicular stomatitis virus infection of splenectomized (SplnX) mice yields a similar number of virus-specific CD8 T cells as control mice. In contrast, vesicular stomatitis virus infection of lymphotoxin-α-deficient knockout (LT-α-KO) mice that lack LNs results in a significant decrease in the total number of virus-specific CD8 T cells [1]. Similarly, intraperitoneal (i.p.) lymphocytic choriomeningitis virus (LCMV) infection of LT-α-KO mice results in a decrease in the total number of virus-specific CD8 T cells in the spleen [2]. Taken together, these data suggest that virus-specific CD8 T cell responses are initiated in LNs following systemic viral infection. However, it is currently unclear which LNs are primarily responsible for initiating the virus-specific CD8 T cell response following a systemic viral infection. In addition, it is currently unknown how events that occur during induction of the CD8 T cell response affect the distribution of antigen-specific memory CD8 T cells in the draining LN following resolution of the infection.

CD8 T cell entry into LNs is dependent on their differentiation status. Naive CD8 T cells express high cell surface levels of both CD62L and CCR7 [3]. The combined expression of these two molecules facilitates CD8 T cell entry into LNs via binding to peripheral node addressin and CCL21, respectively, in the high endothelial venules [3]. Upon activation, naïve CD8 T cells rapidly proliferate and downregulate expression of CD62L. The loss of CD62L expression combined with the upregulation of new adhesion molecules and chemokine receptors facilitates the trafficking of effector CD8 T cells into peripheral tissues [4]. Following pathogen clearance, CD8 T cells undergo contraction and two major subsets of memory CD8 T cells remain: CD62L^−^ effector memory CD8 T cells and CD62L^+^ central memory CD8 T cells. Effector memory CD8 T cells resemble effector CD8 T cells as they lack expression of CD62L and traffic primarily to peripheral tissues. In contrast, central memory CD8 T cells regain expression of CD62L and more efficiently enter the LNs as compared to either effector or effector-memory CD8 T cells [3], [5]. Furthermore, the lack of CD62L cell surface expression on memory CD8 T cells results in ∼90% decrease in their capacity to migrate into peripheral LNs, suggesting that CD62L expression is necessary for efficient entry of memory CD8 T cells into LNs [6].

We demonstrate that the mediastinal LN (MedLN) serves as the primary draining LN following an i.p. LCMV infection. Furthermore, we show that the majority of the LCMV-specific CD8 T cell response is primed in the MedLN, despite other LNs and the spleen acquiring viral antigens during the course of systemic viral spread. These data suggest that the initial draining LN plays a critical role in initiating the virus-specific CD8 T cell response. In addition, we demonstrate that the majority of LCMV-specific memory CD8 T cells in the MedLN are CD62L^−^ for up to 60 days post-infection (p.i). This accumulation of LCMV-specific CD62L^−^ memory CD8 T cells in the MedLN positively correlates with the presence of a sustained germinal center response and increased LN cellularity. Importantly, we demonstrate that the presence of CD62L^−^ effector memory CD8 T cells in the draining LN is not due to the presence of residual virus-derived antigens, but instead due to the preferential recruitment of CD62L^−^ effector memory CD8 T cells. Taken together, these data suggest that very early events that occur during a systemic viral infection profoundly alter the long-term trafficking of virus-specific memory CD8 T cells.

## Results

### The LCMV-specific CD8 T cell response is primarily initiated in the LNs

**Figure 1:**
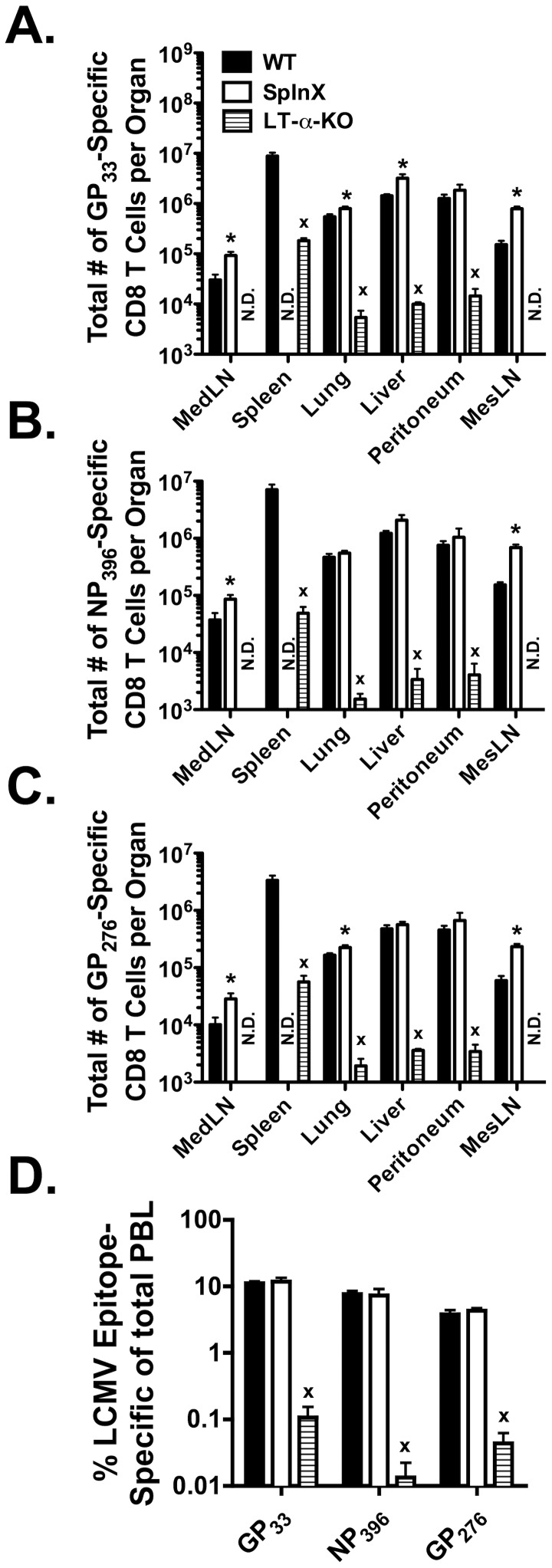
LT-α-KO mice exhibit a dramatically reduced LCMV-specific CD8 T cell response. WT, LT-α-KO and splenectomized mice were infected with LCMV i.p.. The LCMV (A) GP_33_-, (B) NP_396_- and (C) GP_276_-specific CD8 T cell responses from the indicated tissues were measured at day 8 post-LCMV infection via ICS for IFN-γ. (D) GP_33_-, NP_396_- and GP_276_-specific CD8 T cell responses in the peripheral blood. *, significantly greater (*p*<0.05) than WT control mice as determined by Student t-test. ^x^, significantly decreased (*p*<0.05) as compared to WT control mice as determined by Student t-test. Representative data from one of three individual experiments with 3–5 mice per group per experiment. Error bars represent the standard error of the mean. N.D. = none detected.

LCMV infection of mice leads to systemic viral spread with almost all organs supporting virus replication [7]. Previous studies have shown that LT-α-KO mice that lack peripheral LNs exhibit a ∼5-fold decreased LCMV-specific CD8 T cell response as compared to wild-type (WT) mice, suggesting that the majority of the LCMV-specific CD8 T cell response is primed in the LNs [2]. However, LT-α-KO mice also exhibit alteration in splenic architecture [8], thus not completely ruling out a role for the spleen in priming the LCMV-specific CD8 T cell response. Therefore, to confirm the role of the LNs vs. the spleen in priming the LCMV-specific CD8 T cell response, WT, LT-α-KO as well as SplnX mice were infected i.p. with LCMV. Organs were harvested at day 8 p.i. and the total number of LCMV-specific CD8 T cells was assessed by intracellular cytokine staining (ICS) for IFN-γ. Consistent with previous studies [2], Figure 1 demonstrates that LT-α-KO mice exhibit a >10-fold decrease in the total number of LCMV glycoprotein (GP)_33_-specific (Figure 1A), nucleoprotein (NP)_396_-specific (Figure 1B) and GP_276_-specific (Figure 1C) CD8 T cells in the spleen, lung and liver as compared to their WT counterparts. A similar decrease in the frequency of LCMV-specific CD8 T cells was observed in the peripheral blood (Figure 1D). In contrast, there were similar total numbers of LCMV-specific CD8 T cells in the lung, liver and peripheral blood (Figure 1) in SplnX mice as compared to WT mice. Furthermore, there was a significantly (*p*<0.05) greater total number of CD8 T cells of all specificities in the mesenteric LN (MesLN) and MedLN in SplnX mice as compared to WT mice. These data indicate that the spleen is not required to mount a LCMV-specific CD8 T cell response whereas the LNs are necessary for the efficient priming of the CD8 T cell response.

### LCMV viral titers in secondary lymphoid organs early following infection

**Figure 2:**
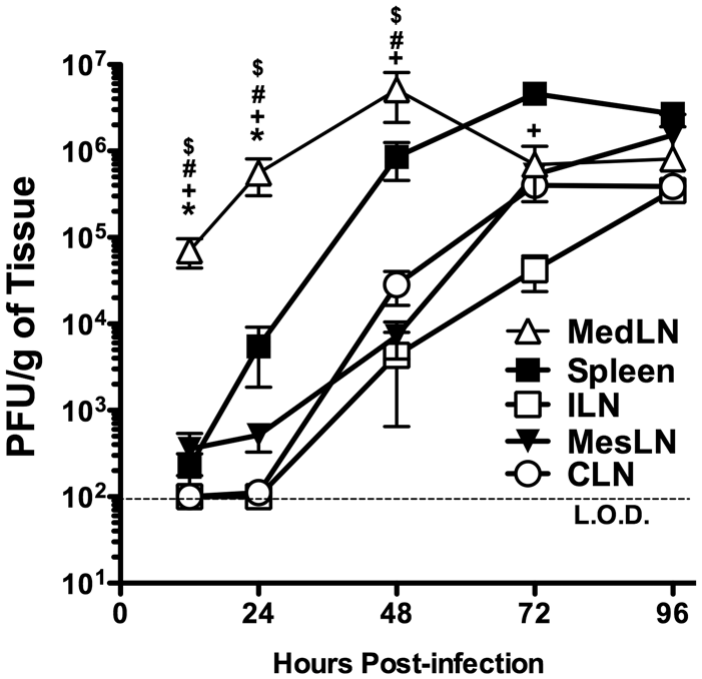
Viral titers in secondary lymphoid tissues early following LCMV infection. C57BL/6 mice were infected with LCMV i.p.. At the indicated times p.i. the spleens, ILNs, MesLNs, CLNs and MedLNs were harvested and assayed for viral titers by plaque assay (A). Data represent pooled data from 3–4 experiments with 3–4 mice per group, except for the ILN and CLN at 0.5 days p.i. where the data represent six mice from two individual experiments. * significantly greater (*p*<0.05) than spleen ^+^, significantly increased (*p*<0.05) as compared to ILN. ^#^ significantly increased (*p*<0.05) as compared to MesLN. ^$^, significantly increased as compared to CLN. All statistical analysis was done by *ANOVA* with a Tukey post-test.

It is currently unclear which specific LN is responsible for initiating the virus-specific CD8 T cell response following an i.p. LCMV infection. A previous study demonstrated that soluble antigens, bacteria, or dyes administered i.p. all drained into the MedLN [9]. Therefore, we hypothesized that the MedLN would serve as the draining LN following an i.p. LCMV infection. Mice were infected i.p. with LCMV and viral titers in the spleen, MedLN, inguinal LN (ILN), MesLN and cervical LN (CLN) were assessed by plaque assay. Figure 2 shows that there is significantly (*p*<0.05) more virus in the MedLN than either the spleen or MesLN at 12 or 24 hours (h) p.i.. Furthermore, there was no virus detected at either 12 or 24 h p.i. in either the ILN or the CLN. However, by 48 h p.i., all tissues examined contained detectable levels of virus. The viral titers in the MedLN peaked at 48 h p.i. and started to decline by 72 h p.i., whereas viral loads in other tissues had either plateaued (i.e. spleen) or continued to increase (i.e. ILN, MesLN and CLN) until 96 h p.i.. Taken together, these data suggest that i.p. infection with LCMV leads to an initial infection of cells within the MedLN.

### Priming of the LCMV-specific CD8 T cell response occurs in the MedLN

**Figure 3:**
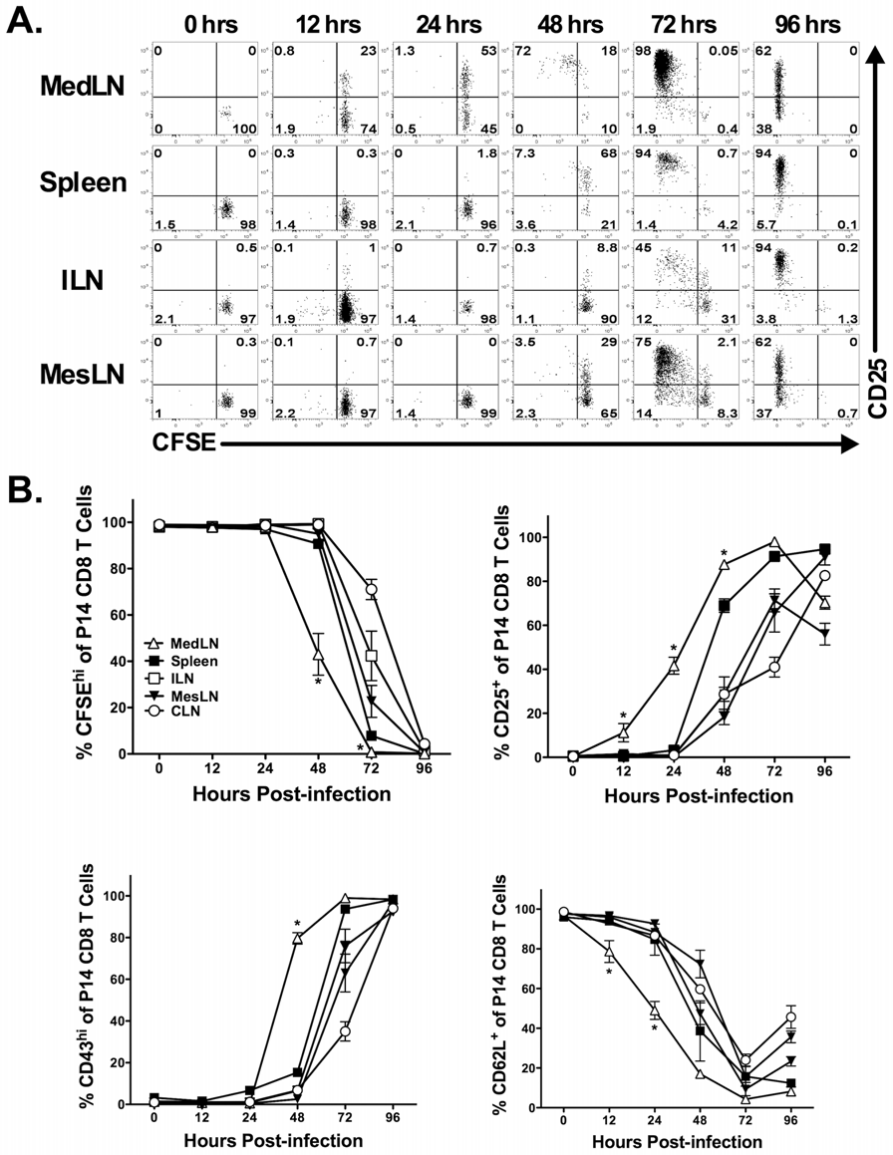
Intraperitoneal infection with LCMV results in rapid antigen display in the MedLN. CFSE-labeled Thy1.1^+^ P14 TCR transgenic CD8 T cells were adoptively transferred into naïve Thy1.2^+^ C57BL/6 mice that were subsequently infected i.p. with LCMV 24 h later. At the indicated times, various organs were harvested and P14 CD8 T cells were assessed for CFSE dilution and expression of activation markers. (A) Representative dot plots showing CFSE dilution profile and CD25 expression. (B) Quantification of CFSE dilution as well as CD25, CD43^glyco^ and CD62L expression. Representative data from one of three individual experiments with three mice at each time point is shown. CD62L data is from two individual experiments with three mice at each time point. *, MedLN is significantly different (*p*<0.05) as compared to all other tissues as determined by *ANOVA* with a Tukey post-test. Error bars represent the standard error of the mean for three mice per group.

The above results indicate that following systemic LCMV infection the infectious virus drains first to the MedLN. We next sought to determine if the presence of infectious virus in the MedLN early following an i.p. infection correlated with initial CD8 T cell priming in the MedLN. We adoptively transferred 2×10^6^ carboxyfluorescein succinimidyl ester (CFSE)-labeled LCMV-specific T cell receptor transgenic P14 CD8 T cells into naïve mice prior to i.p. LCMV infection. At various times p.i., P14 CD8 T cells in the spleen and LNs were monitored for increased CD25 expression as well as proliferation via CFSE dilution. Figures 3A and 3B demonstrate that P14 CD8 T cells upregulate CD25 expression in the MedLN as early as 12 h p.i.. In contrast, we did not observe substantial upregulation of CD25 on P14 CD8 T cells in the spleen, ILN or MesLN until 48 h p.i. (Figure 3A, B). Furthermore, CD8 T cell proliferation occurred initially in the MedLN at 48 h p.i., followed by the spleen, ILN and MesLN at 72 h p.i. (Figure 3A, B). By 96 h p.i. all P14 CD8 T cells in each of the organs examined had proliferated (Figure 3A, B). In addition, downregulation of CD62L and upregulation of CD43^glyco^ on P14 CD8 T cells occurred first in the MedLN at 24 h and 48 h p.i., respectively (Figure 3B).

**Figure 4:**
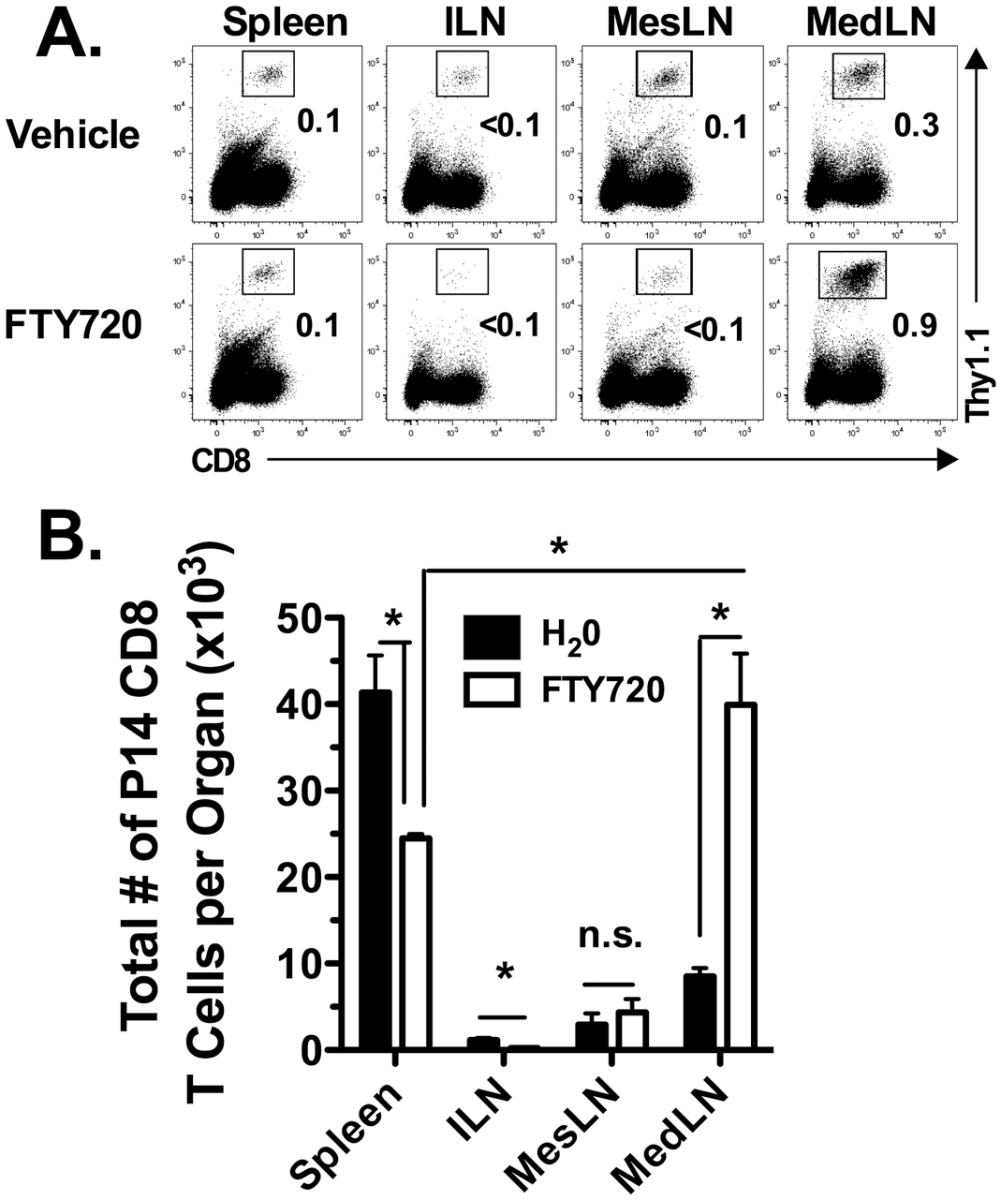
Priming of the LCMV-specific CD8 T cell response occurs in the MedLN. Thy1.1^+^ P14 CD8 T cells were adoptively transferred into naïve Thy1.2^+^ C57BL/6 recipients that were subsequently infected i.p. with LCMV 24 h later. Starting one day after infection, mice were treated i.p. with either 50 µg of FTY720 or vehicle daily. Spleens, ILN, MesLN and MedLN were harvested on day 5 p.i. and analyzed for (A) the frequency of P14 CD8 T cells and (B) the total number of P14 CD8 T cells. *, significantly different (*p*<0.05) as determined by Student t-test. n.s., not significant. Representative data from one of four individual experiments with 3–5 mice per group is shown.

The above results suggest that LCMV-derived antigens are displayed first to naïve CD8 T cells in the MedLN following an i.p. LCMV infection. To examine the role of the MedLN as compared to the non-draining LNs and the spleen in priming the LCMV-specific CD8 T cell response, we adoptively transferred a physiological number (i.e. 2,000) of P14 CD8 T cells into naïve mice one day prior to infection. Starting 24 h p.i., the mice were treated daily with either vehicle (i.e. H_2_O) or the S_1_P receptor agonist FTY720 to trap LCMV-specific CD8 T cells in the LNs. Figure 4 demonstrates that at day 5 p.i. there is a decrease in the frequency (Figure 4A) and a significant decrease (*p*<0.05) in the total number (Figure 4B) of P14 CD8 T cells in the spleen and ILN in FTY720-treated mice as compared to control mice treated with vehicle. There were a similar total number of P14 CD8 T cells in the MesLN in both the control and FTY720-treated mice, suggesting that the MesLN may induce a small proportion of the LCMV-specific CD8 T cell response consistent with the low level of virus in the MesLN early following infection. In contrast to the spleen and ILN, there was a significant increase (*p*<0.05) in the frequency and total number of P14 CD8 T cells in the MedLN of FTY720-treated mice as compared to vehicle-treated control mice (Figure 4). Furthermore, there was a significantly greater (*p*<0.05) number of P14 CD8 T cells in the MedLN of FTY720-treated mice as compared to the spleen of FTY720-treated mice. These data suggest that the initiation of the virus-specific CD8 T cell response occurs primarily in the MedLN early following i.p. LCMV infection.

### Heightened and prolonged presence of germinal center B cells in the MedLN

**Figure 5:**
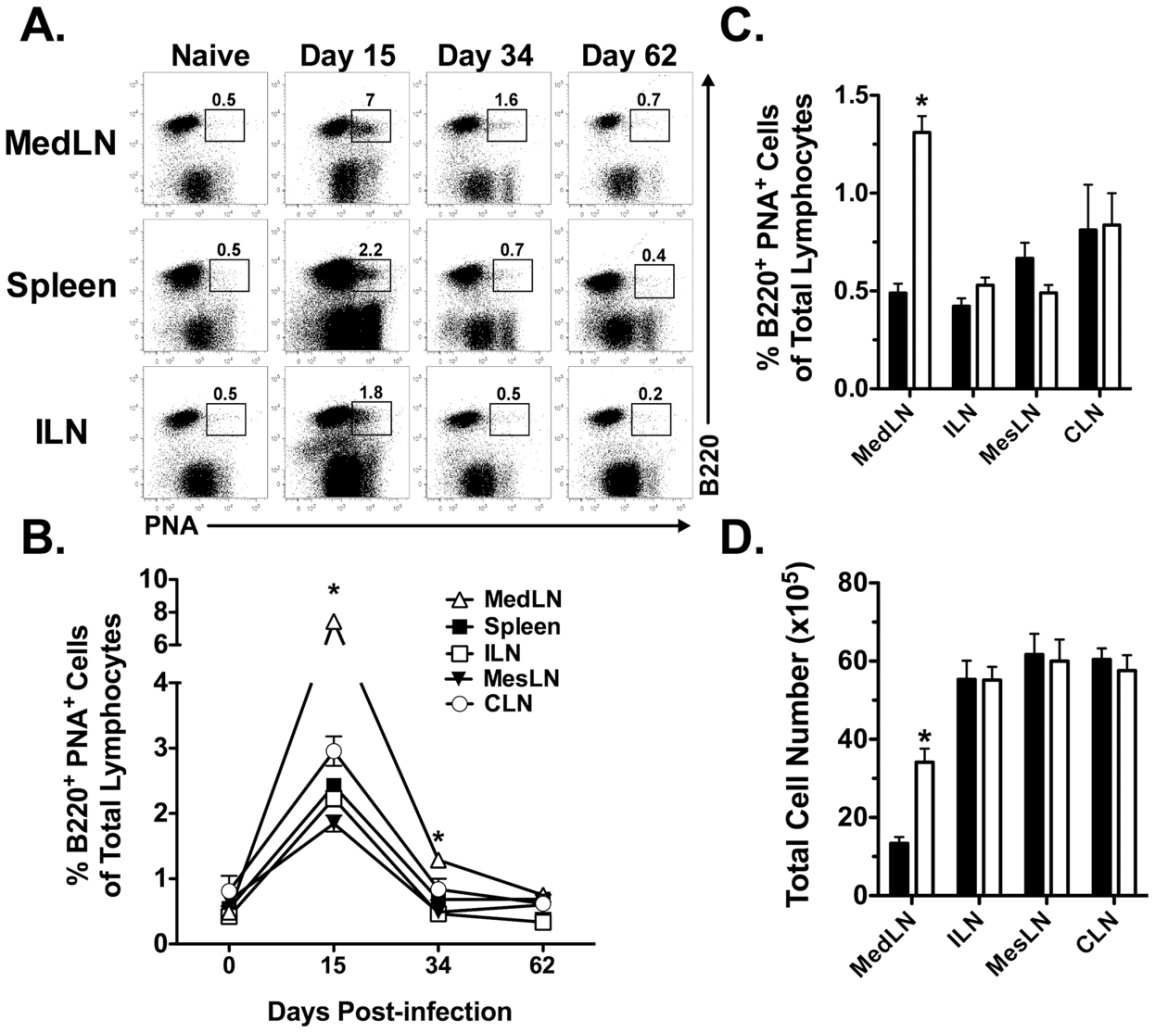
Long-term reactivity of the MedLN following an i.p. infection with LCMV. C57BL/6 mice were infected i.p. with LCMV and at various times following infection the indicated tissues were harvested and analyzed for the presence of GC B cells by B220 and PNA staining. (A) Representative dot plots showing the frequency of GC B cells at various times p.i. (B) Kinetic analysis of GC B cells following an i.p. infection with LCMV. *, MedLN is significantly greater (*p*<0.05) as compared to all other tissues as determined by *ANOVA*. (C) Comparison in the frequency of GC B cells in LNs before or 34 days following LCMV infection. (D) Total cell number in LNs either prior to or 34 days following LCMV infection. *, significantly greater (*p*<0.05) than naïve mice as determined by Student t-test. These data represent more than 4–5 independent experiments, where in some experiments MedLN and CLN were pooled from three mice. Error bars represent the standard error of the mean for greater than four samples per time point.

Following localized immunizations, the draining LN can exhibit altered characteristics such as the presence of germinal center (GC) B cells and increased overall cellularity [10]–[12] identifying it as a reactive LN. Given that the majority of the CD8 T cell response is primed in the MedLN following a systemic LCMV infection, we hypothesized that the MedLN would exhibit a “reactive” phenotype. To test this hypothesis, we examined the presence of GC B cells as a measure of LN reactivity following LCMV infection. The draining MedLN exhibited a significantly increased (*p*<0.05) frequency of GC B cells at days 15 and 34 p.i. as compared to the non-draining LNs (i.e. ILN, CLN and MesLN) and the spleen (Figure 5A, B). However, by day 60 (Figure 5B) and >400 p.i. (data not shown) the frequency of GC B cells was similar between all LNs examined. Furthermore, the frequency of GC B cells was significantly greater (*p*<0.05) at day 34 p.i. only in the MedLN as compared to the corresponding LN in naïve mice (Figure 5C). Additionally, reactive LNs exhibit a prolonged increase in overall cellularity as compared to naïve LNs [12]. Figure 5D demonstrates that there were a greater (*p*<0.05) total number of cells in day 34 p.i. MedLN relative to the MedLN from naïve mice. In contrast, there was no difference (*p*>0.05) in the total cell number between naïve ILN, MesLN or CLN as compared to the same LNs obtained from mice infected 34 days prior with LCMV (Figure 5D). These data demonstrate that following an acute systemic viral infection, the initial draining LN remains “chronically” reactive for an extended period of time.

### CD62L expression pattern on memory CD8 T cells in the reactive and non-reactive LNs

**Figure 6:**
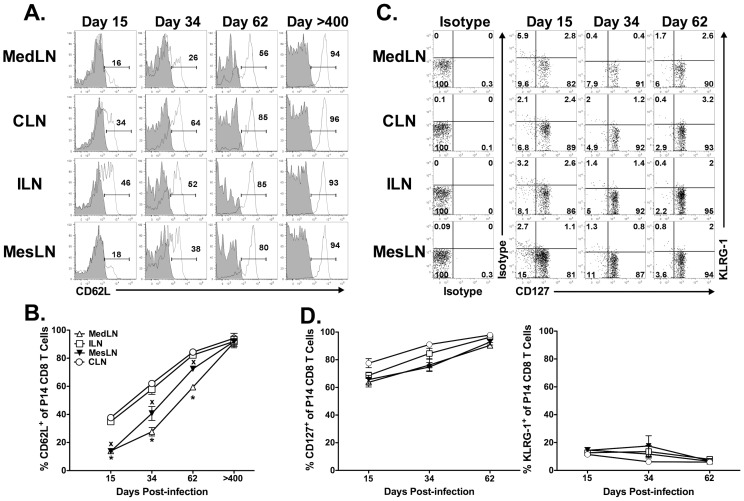
The long-term presence of CD62L^−^ LCMV-specific CD8 T cells in the MedLN. Naïve Thy1.1^+^ P14 CD8 T cells were adoptively transferred into naïve Thy1.2^+^ recipients that were subsequently infected i.p. with LCMV 24 h later. At the indicated times p.i. tissues were harvested and P14 cells were analyzed for expression of CD62L. (A) Representative histograms depicting CD62L staining (open histograms) as compared to isotype control staining (filled histogram). (B) Kinetic analysis of CD62L expression on P14 CD8 T cells. (C), Representative dot plots depicting surface expression of CD127 and KLRG-1 on P14 CD8 T cells in various tissues following LCMV infection. (D) Kinetic analysis of CD127 and KLRG-1 expression of P14 CD8 T cells in the indicated tissues following LCMV infection. *, MedLN is significantly decreased (*p*<0.05) as compared to ILN or CLN. ^x^, MesLN is significantly decreased (*p*<0.05) as compared to ILN or CLN. Combined data representing four independent experiments with 3–4 mice per group is shown. In some experiments, LNs from >day 15 p.i. were pooled and analyzed as a pooled sample.

Previous work has shown that reactive and non-reactive LNs differ in their capacity to attract both memory CD4 and CD8 T cells [12], [13]. Therefore, based on our results demonstrating that the MedLN remains “chronically” reactive following an i.p. LCMV infection, we questioned if the trafficking of memory CD8 T cells into the MedLN would be altered. Given the importance of CD62L for entry of T cells into LNs, we compared the expression of CD62L on P14 cells in the MedLN vs. other LNs. The reactive MedLN exhibited a reduced frequency of CD62L^+^ P14 CD8 T cells at days 15, 34 and 62 p.i. as compared to the non-reactive LNs (i.e. ILN and CLN) (Figure 6A). However, by day >400, all LNs exhibited a similar frequency of CD62L^+^ P14 CD8 T cells. The chemokine receptor CCR7 represents another important molecule involved in T cell entry into the LN. In contrast to CD62L, the frequency of CCR7^+^ P14 CD8 T cells was similar between the MedLN and the other LNs at day 34 p.i. (Figure S1). In addition, the frequency of CD62L^+^ P14 CD8 T cells in the MesLN, a LN that has been previously shown to utilize α_4_β_7_ in addition to CD62L for CD8 T cell entry [14]–[16], was similar to that of the MedLN at all time points examined (Figure 6A, B). Consistent with a role for α_4_β_7_ in facilitating entry of T cells into the MesLN, we observed an increased frequency of β_7_^+^ P14s in the MesLN as compared to the MedLN, ILN and CLN (Figure S2). Although the majority of P14 CD8 T cells in the MedLN were CD62L^−^ for ∼60 days following infection, the expression levels of two other memory-associated molecules (e.g. CD127^hi^ and KLRG-1^lo^) were remarkably similar between P14 cells in the MedLN as compared to the P14 cells in the ILN and CLN at virtually all times following LCMV infection (Figure 6C, D). These data argue against retention of effector CD8 T cells in the MedLN long-term following viral infection and rather suggest that virus-specific memory CD8 T cells that enter the MedLN>15 days following an i.p. infection with LCMV do not require CD62L.

### Accumulation of CD62L^−^ effector memory CD8 T cells in the reactive LN is not due to residual antigen presentation

**Figure 7:**
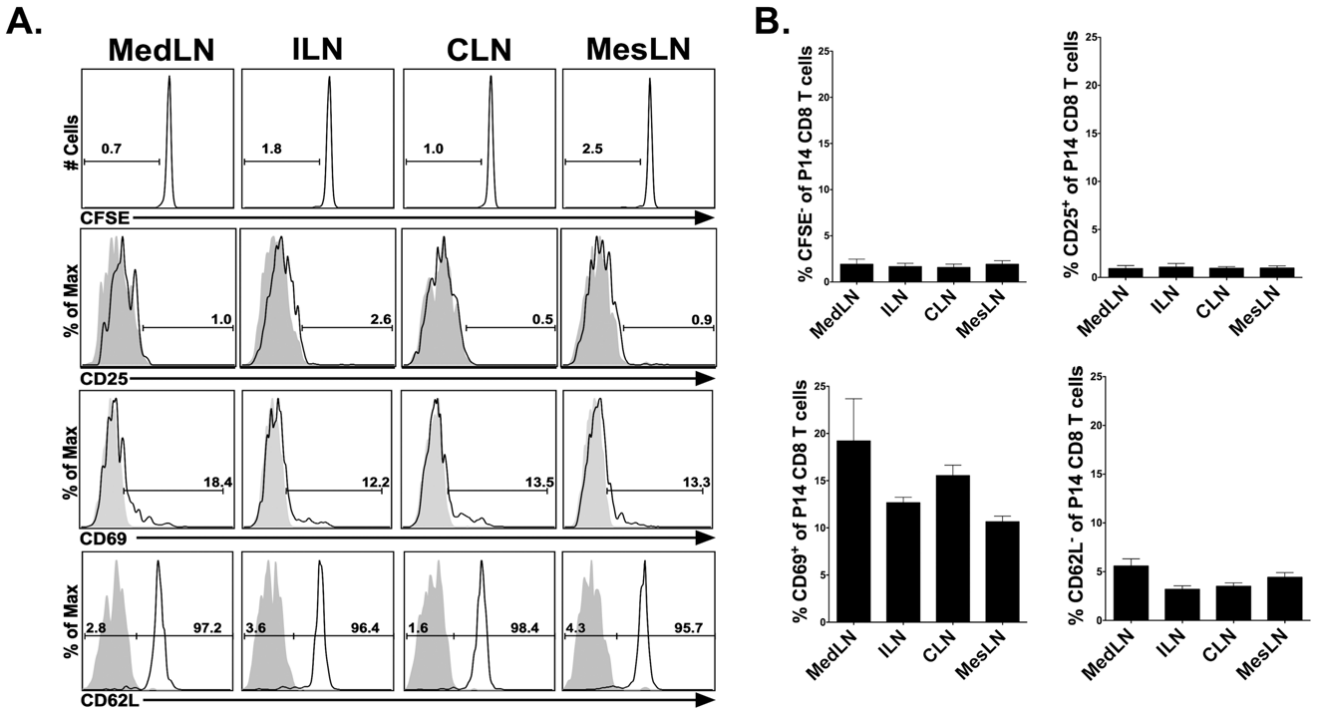
Naïve cells are not activated by residual antigen in the draining LN. CFSE-labeled naïve P14 CD8 T cells were transferred into day 34 LCMV-infected mice. The indicated LNs were harvested 6 days post-transfer and the transferred cells were analyzed for CFSE dilution, and expression of CD25, CD69 and CD62L expression. (A) Representative dot plots showing CFSE, CD25, CD69 and CD62L profiles gated on naive P14 CD8 T cells. (B) Frequency of CFSE^hi^, CD25^+^, CD69^+^ and CD62L^+^ P14 CD8 T cell in the respective LNs. Combined data from two individual experiments with four mice per group. Error bars represent the standard error of the mean.

**Figure 8:**
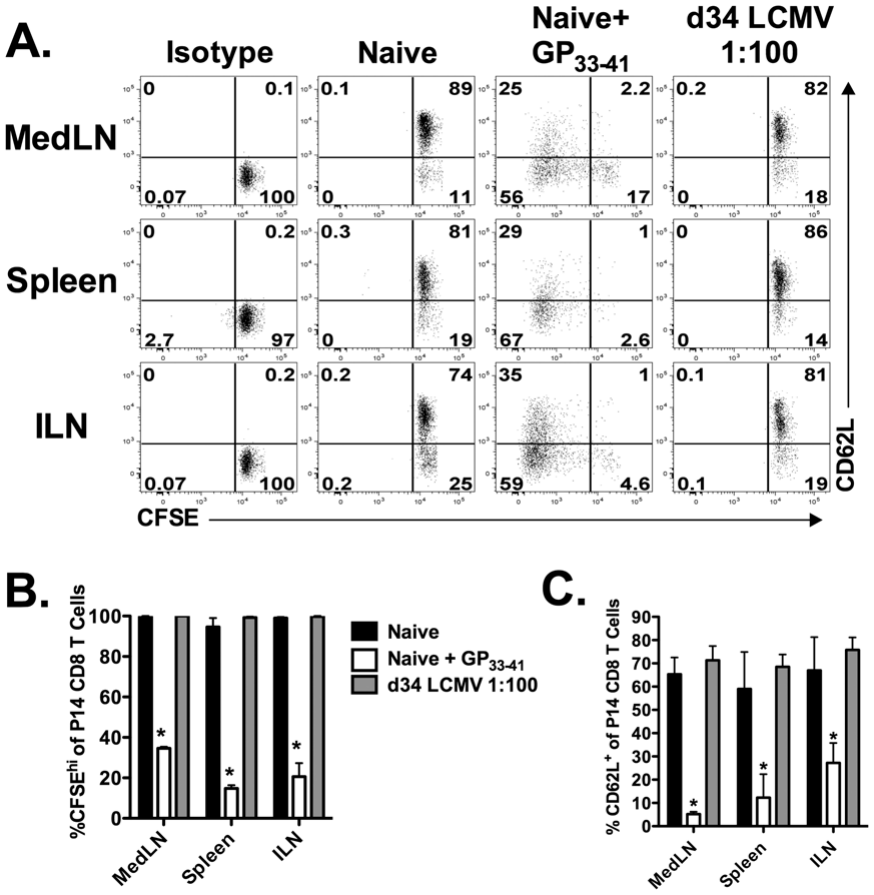
No prolonged presence of viral antigens in the MedLN following an i.p. LCMV infection. CFSE-labeled memory P14 CD8 T cells were co-cultured with single-cell suspensions of either splenocytes or LN cells from naïve (1∶100 memory P14∶spleen/LN cell) and day 34 LCMV-infected mice (1∶100 memory P14∶spleen/LN cell). Memory CFSE-labeled P14 cells were also cultured with either naïve splenocytes or LN cells pulsed with 1 µM GP_33–41_ peptide as a positive control. Cells were co-cultured for 3 days and subsequently analyzed for CFSE dilution, and either CD25 or CD62L expression. (A) Representative dot plots showing CD62L and CFSE profiles gated on memory P14 CD8 T cells. (B) Frequency of CFSE^hi^ P14 CD8 T cells after 3 days of co-culture with either splenocytes or LN cells from either naïve or LCMV-infected mice as described above. (C) Frequency of CD62L^+^ P14 CD8 T cells after 3 days of co-culture with either splenocytes or LN cells from naïve or LCMV-infected mice as described above. *, significantly decreased (*p*<0.05) as compared to P14 memory cells co-cultured with either control naïve splenocytes or LN cells as determined by Student t-test. Data are representative of three individual experiments with 3–4 mice per group. Error bars represent the standard error of the mean.

**Figure 9:**
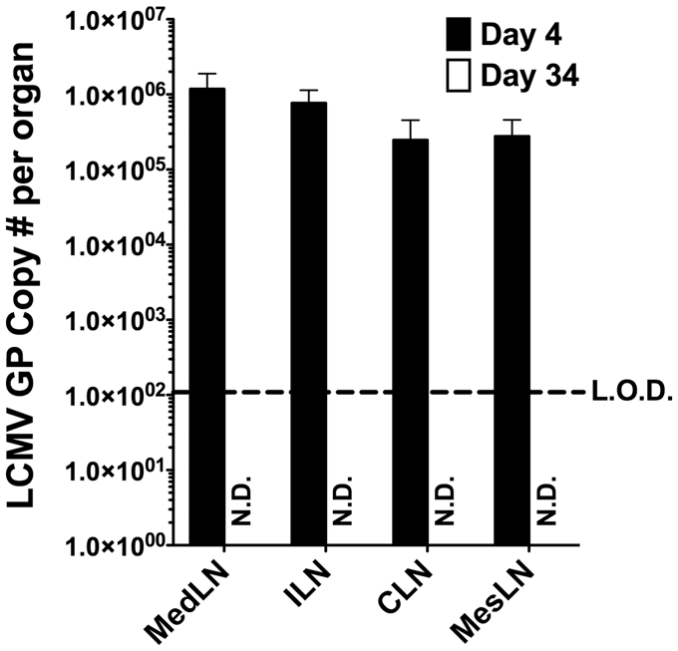
No residual viral RNA in the draining LN following an i.p. LCMV infection. RNA was isolated from naïve, day 4 and day 34 LCMV infected mice, and quantified by RT-PCR. N.D. not detected. Combined data from two individual experiments with four mice per group. Error bars represent the standard error of the mean.

One potential explanation for the decreased frequency of CD62L^+^ P14 CD8 T cells in the MedLN as compared to either the ILN or the CLN is the presence of residual virus-derived antigen. Persistent antigen in the MedLN could cause reactivation of memory P14 cells resulting in either the down-regulation or the cleavage of CD62L. Work by Khanna et al. demonstrates that residual antigen is present within the MedLN for up to 30 days following an acute pulmonary influenza virus infection and that this antigen is capable of activating newly recruited CD8 T cells [17]. The reactive MedLN contained an increased frequency of CD25^+^ and CD69^+^ memory P14 cells as compared to the non-reactive LNs 34 days following LCMV infection (Figure S3A, B). This increased frequency of CD25^+^ and CD69^+^ memory P14 cells in the MedLN could be the result of continued antigen presentation in this LN due to the presence of persisting antigen. Therefore, to determine if residual antigen persists within the reactive MedLN, naïve P14 CD8 T cells were CFSE-labeled and subsequently transferred into day 34 LCMV-immune mice. MedLN, ILN, MesLN and CLNs were harvested 6 days post-transfer and the activation status of the transferred P14s was analyzed. The transferred P14s did not exhibit upregulation of the activation markers CD25 and CD69 following transfer (Figure 7A, B). Additionally, the transferred cells did not dilute CSFE expression or downregulate CD62L expression (Figure 7) indicating that no residual antigen is present within the MedLN (or within any other LN) of a mouse infected with LCMV 34 days prior. The above experiments were performed with naïve P14 CD8 T cells because previous data has suggested that transferred memory cells may not be able to fully access antigen-bearing dendritic cells (DCs) [17]. However, it is well established that memory CD8 T cells display an increased sensitivity to antigen as compared to naïve CD8 T cells [18], [19]. Therefore, to further test if residual antigen is present within the MedLN using memory cells, we harvested the MedLNs, ILNs and spleens from mice infected 34 days prior with LCMV. Mononuclear cells from these tissues were cultured *in vitro* with CFSE-labeled memory P14 CD8 T cells that had been isolated from the spleens of LCMV-immune mice. After 3 days in culture we examined the expression of CD62L and the dilution of CFSE on the memory P14 CD8 T cells. Memory P14 CD8 T cells did not divide nor downregulate cell surface expression of CD62L when cultured with cells isolated from the spleen, MedLN, CLN or ILN of either naïve or day 34 LCMV-infected mice (Figure 8 A–C). However, the memory P14 CD8 T cells did both divide and downregulate CD62L when cultured with tissue-derived mononuclear cells pulsed with GP_33–41_ peptide. As an additional test for the presence of residual antigen, we assessed the levels of the LCMV GP by RT-PCR. LCMV GP was readily detectable by RT-PCR in all LNs examined at day 4 p.i. In contrast, no detectable LCMV GP was present within the MedLN at day 34 p.i. as determined by RT-PCR (Figure 9). Taken together, these data suggest that residual LCMV-derived antigen is not responsible for the decreased frequency of CD62L^+^ P14 memory CD8 T cells in the MedLN as compared to the ILN or CLN.

### Increased frequency of CD62L^−^ effector memory CD8 T cells in the reactive LN is not unique to the MedLN

**Figure 10:**
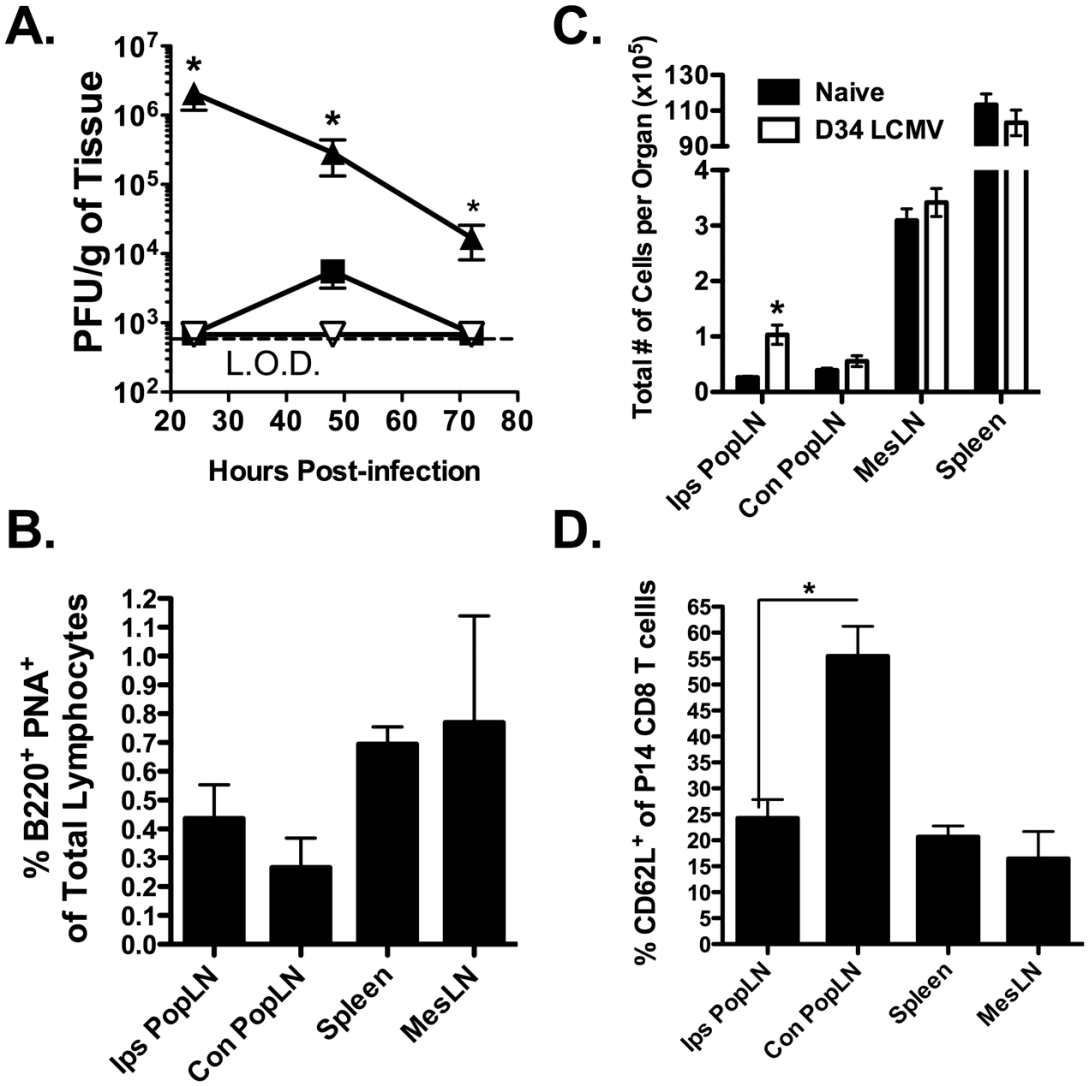
High frequencies of CD62L^−^ LCMV-specific CD8 T cells in the draining LN is not unique to the MedLN. C57BL/6 mice were infected in the footpad with LCMV. (A) Viral titers in the Ips PopLN (solid upwards triangle), Con PopLN (downwards triangle) and spleen (solid square) were measured via plaque assay at 24, 48 and 72 h p.i.. *, Ips PopLN is significantly greater (*p*<0.05) than all other as determined by *ANOVA*. (B and D) Naïve Thy1.1^+^ P14 CD8 T cells were adoptively transferred into naïve Thy1.2^+^ recipients and infected via the footpad with LCMV the following day 34–40 p.i., the indicated tissues were harvested and assessed for the frequency (B) GC B cells (as described in Figure 5) and (D) CD62L^+^ P14 CD8 T cells. (C) The total number of cells in each LN or spleen was assessed in naïve mice or in mice previously infected (day 34–40) with LCMV via the footpad. (B and C) *, Day 34 Ips PopLN is significantly greater (*p*<0.05) than naïve Ips PopLN determined by *ANOVA*. (D) *, Ips PopLN is significantly lower (*p*<0.05) than Con PopLN as determined by *ANOVA*. Combined data from 2–3 individual experiments with four mice per group. Error bars represent the standard error of the mean.

Figure 6 demonstrated an increased frequency of CD62L^−^ LCMV-specific memory CD8 T cells in the reactive MedLN as compared to the non-reactive LNs at day 34 following an i.p. LCMV infection. The MedLNs are similar to the MesLNs of the gut in that they both drain tissues that are constantly inflamed. The gut is constantly exposed to foreign antigen and is inhabited by commensal bacteria. The lung is similar in that it is also continuously exposed to foreign antigens. Thus, it is possible that these LNs share a similar CD62L-independent trafficking mechanism. To address this possibility, we infected mice via the footpad with LCMV. This route of infection is commonly used to direct antigens to the popliteal LN (PopLN) [20], [21]. Early after footpad infection, we examined both PopLNs as well as the spleen to ensure preferential infection of the ipsilateral PopLN (Ips PopLN). Figure 10A shows that at 24 h p.i., only the Ips PopLN contained replicating virus, indicating that the Ips PopLN is the initial draining LN. The spleen contained a small amount of replicating virus only at 48 h p.i. and we were unable to detect virus within the Con PopLN at any time points following footpad infection (Figure 10A). To determine if priming the LCMV-specific CD8 T cell response in the draining Ips PopLN resulted in chronic reactivity of this LN, we examined the presence of GC B cells and total cellularity at days 34–40 p.i.. Although the Ips PopLN did not exhibit a heightened/prolonged presence of GC B cells following footpad infection as compared to either the spleen or other LNs examined (Figure 10B), there were significantly (*p*<0.05) more total cells in the LCMV-immune Ips PopLN as compared to its naïve counterpart (*p*<0.05; Figure 10C) suggesting that the Ips PopLN is reactive. Next, we wanted to determine if a decreased frequency of CD62L^+^ LCMV-specific memory CD8 T cells is present in the reactive Ips-PopLN as compared to the non-reactive Con PopLN. Thirty-four to forty days following footpad infection, the Ips PopLN, Con PopLN, MesLN and spleen were harvested and examined for the presence of CD62L^+^ LCMV-specific memory CD8 T cells. Figure 10D shows a similar low frequency of CD62L^+^ LCMV-specific memory CD8 T cells in the Ips PopLN, MesLN and spleen. However, there was a significantly (*p*<0.05) higher frequency of CD62L^+^ LCMV-specific CD8 T cells in the Con PopLN (Figure 10D). These data suggest that the MedLN does not uniquely attract CD62L^−^ CD8 T cells but rather, this is a property of draining LNs that initiate the virus-specific CD8 T cell response. Furthermore, these data suggest that the prolonged presence of GC B cells is not required for the preferential recruitment of CD62L^−^ memory CD8 T cells.

### CD62L^−^ effector memory CD8 T cells preferentially enter the reactive LN

**Figure 11:**
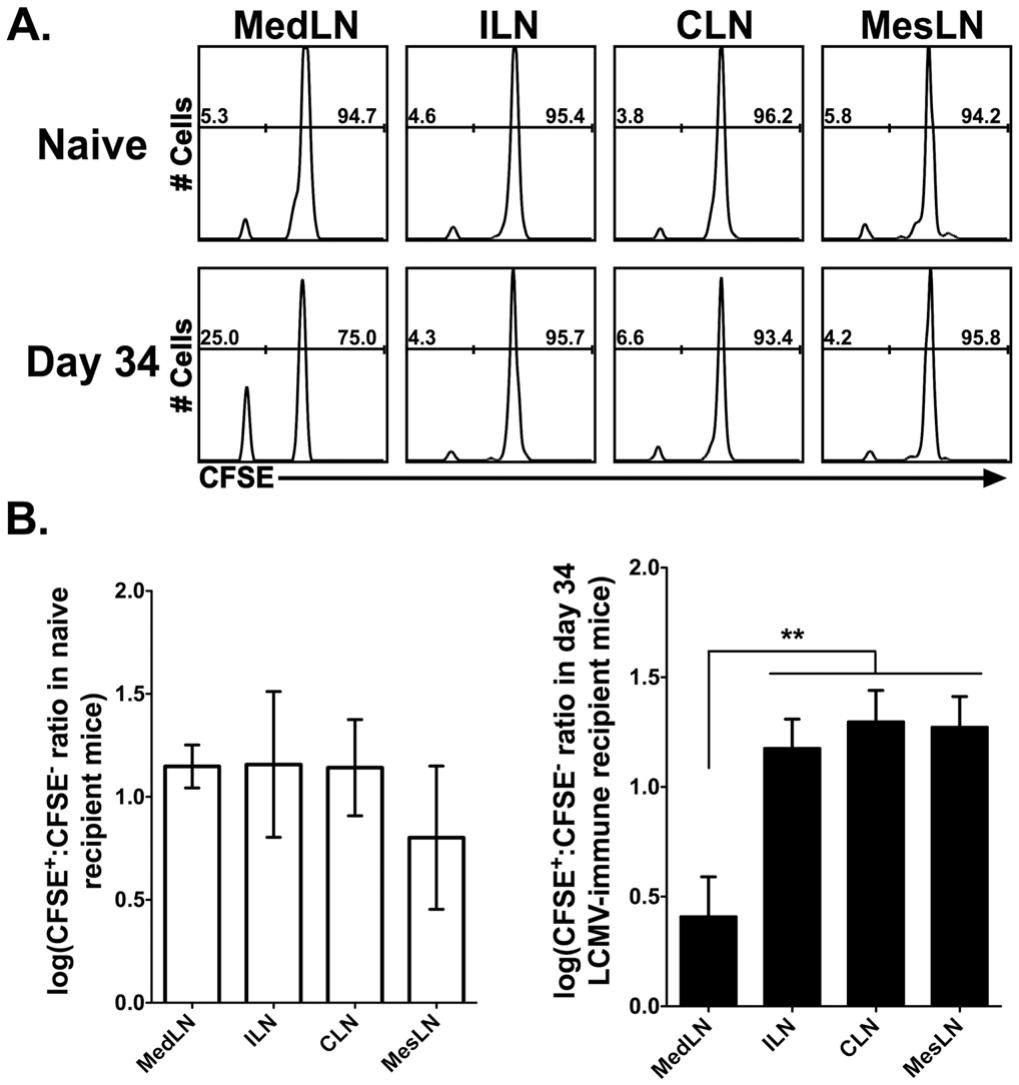
CD62L^−^ effector memory CD8 T cells preferentially traffic to the draining MedLN. CD62L^+^ and CD62L^−^ memory P14 cells were isolated from LCMV-immune mice. CD62L^+^ cells were CFSE-labeled and mixed with unlabeled CD62L^−^ P14 cells and transferred into either naïve or day 34 LCMV infected mice. 72 h post-transfer, LNs were harvested and transferred cells (CD8^+^Thy1.1^+^) were examined for their ratios in naïve mice or day 34 LCMV-infected mice. (A) Representative histograms from naïve (top) and day 34 LCMV-infected (bottom) mice is shown from one of three experiments with 5 mice total per group. (B) Graphs depicting combined data from all experiments presented in a logarithmic scale. Na?¨ve mice are shown the open bars and day 34-LCMV mice are shown in the closed bars. **, MedLN is significantly lower (*p*<0.01) than the ILN, CLN and MesLN as determined by *ANOVA*.

Guarda et al. previously demonstrated that CD62L^−^ effector and effector memory CD8 T cells accumulate in the reactive LN as compared to the non-reactive LNs following DC immunization in the footpad [13]. To determine if preferential recruitment of CD62L^−^ effector memory CD8 T cells occurs following a systemic viral infection, sorted memory CFSE-labeled CD62L^+^ and unlabeled CD62L^−^ P14 CD8 T cells were co-transferred into either naïve mice or day 34 LCMV-infected mice. Seventy-two h post-transfer, the reactive MedLNs and non-reactive ILNs and CLNs were harvested and the ratio of CFSE-labeled cells was examined. The CD62L^−^ memory CD8 T cells (CFSE^−^) exhibited an enhanced capacity to enter the reactive MedLN as compared to the non-reactive LNs in a day 34 LCMV-infected mouse (Figure 11A and B). Importantly, the MedLN of naïve mice displayed a similar ratio of transferred CFSE^+^∶CFSE^−^ cells as compared to the ILNs and the CLNs (Figure 11). These data indicate that in the setting of a systemic viral infection, the initial draining LN demonstrates an increased capacity to recruit CD62L^−^ effector memory CD8 T cells for an extended time following resolution of the infection.

## Discussion

Much is known about the priming of CD8 T cell responses following localized infection by viral pathogens. In these studies, viral antigen is largely restricted to the infected tissue and some of this antigen is transported to the tissue-draining LN, either by DCs or through the lymph, to prime the virus-specific CD8 T cell response [22], [23]. However, the process by which this occurs following a systemic viral infection where viral antigen is not restricted to a single tissue or draining LN is currently unclear. Our data clearly demonstrates that although all of the LNs examined eventually harbor replicating virus, only the immediate draining MedLN was responsible for priming the majority of the virus-specific CD8 T cell response following an i.p. LCMV infection (Figures 3 and 4). In addition, the draining LN remained chronically reactive (Figure 5) and exhibited a profound impact on the trafficking of memory CD8 T cells, allowing the entry of CD62L^−^ effector memory CD8 T cells (Figure 6). Taken together, these data suggest an intimate link between events that occur early during CD8 T cell priming in the draining LN and how this affects the entry of memory CD8 T cell subsets into the initial draining LN long-term following infection.

Several studies have demonstrated the presence of residual antigen in the priming LN following a localized infection by a number of viral pathogens [17], [24], [25]. For example, Khanna et al [17] showed that there was a high frequency of CD62L^−^ virus-specific CD8 T cells in the MedLN 30 days following an intranasal influenza virus infection [17]. Their studies indicate that the high frequency of CD62L^−^ CD8 T cells in the MedLN is due to long-term depots of influenza virus antigen that constantly stimulate CD8 T cells and cause the downregulation of CD62L. Consistent with this notion, transferred naïve, but not memory, influenza virus-specific CD8 T cells upregulate activation markers such as CD69 and PD-1 [17], [24]. These data indicated that the increased presence of CD62L^−^ memory CD8 T cells in the draining MedLN following LCMV infection may be due to residual antigen within this LN. However, we did not observe any signs of activation (i.e. CFSE-dilution, CD25 upregulation, CD69 upregulation or CD62L downregulation) of naïve P14s upon transfer into day 34 LCMV-infected mice (Figure 7). Furthermore, we co-cultured LCMV-specific memory CD8 T cells with MedLN-derived single-cell suspensions to make antigen available to all cells [17] and we were unable to detect the presence of LCMV-derived antigens in the MedLN (Figure 8). These data suggest that neither the long-term reactivity of the MedLN nor the lack of CD62L expression on CD8 T cells is due to the persistence of viral antigen. However, this assay may not be sensitive enough to detect very low levels (<1 pM) of antigen on a small number of DCs or other antigen presenting cells and does not rule out very low-level persistence of antigen. To utilize a more sensitive assay for LCMV detection [26], [27], we utilized RT-PCR and were unable to detect any residual LCMV GP at day 34 p.i. (Figure 9). Thus taken together, our data indicates that residual LCMV-derived antigen is likely not responsible for either the long-term reactivity of the MedLN or the increased frequency of CD62L^−^ effector memory CD8 T cells in the MedLN. Our results are consistent with a recent study by Takamura et al [28] demonstrating that mice infected i.p. with Sendai virus generate virus-specific memory CD8 T cells that are unable to recognize residual antigen in the draining LN as compared to CD8 T cells generated following an intranasal infection.

Another explanation for the increased frequency of CD62L^−^ effector memory CD8 T cells in the draining LN could be due to the preferential recruitment of these cells. Studies using localized DC immunizations in the footpad demonstrated that transferred CD62L^−^ effector and effector memory CD8 T cells entered the draining LN at an increased propensity as compared to the non-draining LN [13]. In concordance with the above study, we demonstrate that the increased frequency of CD62L^−^ CD8 T cells in the draining LN following a systemic LCMV infection is due to the preferential trafficking of CD62L^−^ effector memory CD8 T cells as compared to the non-draining LNs (Figure 11). These data indicate that although LCMV induces a systemic viral infection in which viral replication occurs in virtually all of the secondary LNs, only the initial draining LN remains reactive and allows preferential access for CD62L^−^ effector memory CD8 T cells. However, the mechanism that accounts for the preferential recruitment of CD62L^−^ effector memory CD8 T cells remains unclear. Guarda et al [13] demonstrated that both CD62L^−^ effector and effector memory CD8 T cells utilize the chemokine receptor CXCR3 to enter reactive LNs [13]. Interestingly, we observed that greater than 90–95% of the LCMV-specific CD8 T cells in the MedLN express CXCR3 (data not shown). However, we do not observe any significant difference in the expression of the CXCR3 chemokine ligands CXCL9 and CXCL10 via either RT-PCR or ELISA (data not shown), suggesting that these cells may utilize other means of entry into the MedLN other than CXCR3. Martin-Fontecha et al demonstrated that CD62L^−^ effector memory CD4 T cells could enter long-term/chronic reactive LNs in a CD62P/PSGL-1 (P-selectin glycoprotein ligand 1)-dependent manner [12]. Recent studies have also shown a role for PSGL-1 in the migration of activated T cells and other leukocytes into LNs [29], [30]. In preliminary experiments, we observed that greater than 90–95% of the LCMV-specific CD8 T cells in the MedLN expressed the CD62P ligand PSGL-1 (data not shown). Other studies report that the activated glycoform of CD43 (CD43^glyco^) can play a role in leukocyte adhesion to tissue endothelial cells that express E-selectin (CD62E) which in some scenarios can also be expressed on the high endothelial venules of reactive LNs [31]–[34]. We have also observed that LCMV-specific CD8 T cells in the MedLN express higher levels of CD43^glyco^ as compared to LCMV-specific CD8 T cells in the peripheral blood or spleen (data not shown). These data suggest that LCMV-specific CD8 T cells may enter reactive LNs via a PSGL-1-or CD43-dependent manner.

Recent work has suggested that T cells can be “imprinted” upon priming to preferentially traffic to the tissue in which the antigens originated. For instance, CD8 T cells primed in either the gut draining LNs (e.g. MesLN) or the Peyer's Patches are imprinted with the gut homing integrin α_4_β_7_ [14], [15] whereas T cells primed in peripheral LNs do not express α_4_β_7_, but instead express the α_4_β_1_ integrin which plays a role in homing to other inflamed tissues (i.e. the skin and lung) [35]. It is possible that CD8 T cells primed in the MedLN are “imprinted” in this manner to preferentially return to the MedLN. Figure 3 demonstrates that LCMV-specific CD8 T cells in the MedLN 4 days following an i.p. infection with LCMV maintain a CD62L^−^ phenotype. In comparison, LCMV-specific CD8 T cells in other LNs (i.e. the ILN and CLN) largely regain CD62L expression at day 4 post-LCMV infection. These data correlate with what we observed >30 days p.i. where there is a higher frequency of CD62L^+^ LCMV-specific CD8 T cells in the ILN and CLN as compared to the MedLN. Taken together, these data may provide evidence for “imprinting” of LCMV-specific CD8 T cells primed in the MedLN to return to the MedLN in a CD62L-independent manner following resolution of the infection. However, it is important to note that this imprinting is not due to expression of α4β7 as we observed a similar frequency of β_7_-expressing memory CD8 T cells within the draining MedLN as we do in the non-draining LNs (Figure S2).

It is unclear if there is an advantage to have CD62L^−^ virus-specific effector memory CD8 T cells in the draining LN long-term after infection. Khanna et al [17] demonstrated that influenza-specific CD8 T cells in the MedLN express activation markers (i.e. CD69 and PD-1) that are often co-expressed with granzyme B after migration into inflamed tissues. Cells with primed effector function may be maintained in the draining LN long-term to provide a first line of defense against pathogens that replicate in secondary lymphoid organs in order to protect against secondary infection via the same route of infection. Figure 2 shows that the MedLN is the first place where replicating virus can be detected following an i.p. infection with LCMV. These data suggest that like other tissues, the draining LN may serve as a reservoir for effector-memory CD8 T cells to serve as local guardians against re-infection.

Overall, our data demonstrates that following a systemic viral infection, the vast majority of virus-specific CD8 T cells are primed within the initial draining LN. Furthermore, our data demonstrates that the long-term trafficking of virus-specific memory CD8 T cells is altered in the draining LN as compared to the non-draining LNs for an extended period of time following resolution of infection, preferentially recruiting CD62L^−^ effector memory CD8 T cells. Our data provides important insight into how vaccines may be manipulated to improve initial CTL responses to particular pathogens. The route of immunization can be controlled to target specific LNs that may be involved in responding to viral infections. For example, either intranasal or i.p. immunization against influenza virus may provide a long-term resident population of effector memory CD8 T cells in the lung draining LNs that are better suited to elicit effector functions after live virus infection [36], [37]. Furthermore, other mucosal immunization routes may be utilized to enhance activated CD8 T cells in the LNs that drain the vaginal tract to protect against either HIV-1 or HSV infection.

## Materials and Methods

### Ethics statement

All experimental procedures utilizing mice were approved by the University of Iowa Animal Care and Use Committee. The experiments performed in this study were done under strict accordance to the Office of Laboratory Animal Welfare guidelines and the PHS Policy on Humane Care and Use of Laboratory Animals.

### Virus and mice

The Armstrong strain of LCMV was a gift from Raymond Welsh (University of Massachusetts Medical School, Worcester, MA) and was propagated in baby hamster kidney cells (American Type Culture Collections; ATCC, Manassas, VA). C57BL/6NCr Thy1.2^+^ mice were obtained from the National Cancer Institute (Frederick, MD). SplnX C57BL/6NCr mice were obtained from the National Cancer Institute and the splenectomy was performed at Charles River Laboratories (Wilmington, MA). SplnX mice were rested for greater than one month following splenectomy prior to LCMV infection. LT-α-KO mice were a gift from Dr. John Harty (University of Iowa, Iowa City, IA). All mice were age-matched and infected i.p. with 5×10^4^ plaque forming units (PFU) of LCMV. T cell receptor transgenic P14 CD8 T cells (specific for the LCMV GP_33–41_ epitope) were isolated from either the spleen or peripheral blood of Thy1.1^+^ P14 mice. CFSE labeling of P14 CD8 T cells was performed by incubating 10^7^ splenocytes/ml from P14 mice for 10 minutes at 37°C in the presence of 5 µM CFSE. CFSE-labeled cells were washed twice with RPMI 1640 containing 10% fetal calf serum and twice with sterile PBS. In some experiments, LCMV-infected mice were treated from days +1 to +4 with 50 µg of FTY720 (Cayman Chemical Co., Ann Arbor, MI) in sterile, endotoxin-free H_2_O. Control mice were administered H_2_O.

### Plaque assay

Mice were infected with LCMV and at various times p.i., organs were harvested and placed in sterile, serum-free RPMI 1640. Spleens and LNs were disrupted using a tissue homogenizer (Ultra-Turrax T25, IKA, Wilmington, NC) and tissue homogenates were subsequently centrifuged at 2000 rpm for 10 min. Cell-free supernatants were collected and snap-frozen in liquid nitrogen prior to storage at −80°C. Samples were thawed and virus titers were determined by plaque assay on Vero cells.

### Mononuclear cell isolation and ICS

Tissues were harvested and mononuclear cells were obtained from the spleen and LNs by pressing the organs between the ends of frosted slides. In some experiments, the spleen and LNs were digested prior to mononuclear cell isolation to ensure maximal liberation of cells. Spleens and LNs were diced and placed in 5 or 1 ml, respectively, of Hank's balanced salt solution supplemented with 125 U/ml of collagenase type II (Invitrogen), 60 U/ml of DNAse I type II (Sigma-Aldrich, St. Louis, MO) and incubated at 37°C for 30 min followed by disruption with frosted glass slides. In experiments where lungs and livers were harvested the mice were first perfused with 20 ml of sterile saline and the tissues were subsequently pressed through a wire mesh screen (Cellector, Bellco Glass, Inc., Vineland, NJ). Blood was collected from isoflourane anethsitized mice by eye bleed into 4% (w/v) sodium citrate and red blood cells were lysed with NH_4_Cl. Peptides corresponding to the LCMV CD8 T cell epitopes GP_33–41_, NP_396–404_ and GP_276–286_ were purchased from Biosynthesis Inc. (Lewisville, TX). To enumerate the number of LCMV-specific CD8 T cells, mononuclear cells from the spleen, LNs and livers were stimulated *in vitro* in the presence of 1 µM peptide and 10 µg/ml brefeldin A (Sigma-Aldrich) for 5 h at 37°C. Previous work from our laboratory has shown that mononuclear cells isolated from the lung and peripheral blood require stimulation by exogenous antigen presenting cells coated with peptide to accurately enumerate the number of antigen-specific cells in these locations [38]. Lung and peripheral blood were stimulated with EL4 cells (American Type Culture Collection, Manassas, VA) coated with 1 µM peptide in the presence of brefeldin A for 5 h at 37°C. Cells were subsequently stained for cell surface CD4, CD8, Thy1.2 and intracellular IFN-γ as previously described [39]. In some experiments, cells from LNs and spleens were stained with fluorescein isothiocyanate-conjugated peanut agglutinin (Vector Laboratories, Burlingame, CA), B220 (eBioscience) and CD19 (eBioscience) for the detection of germinal center B cells.

### In vitro antigen detection assay

Mice were infected with 5×10^4^ PFU of LCMV i.p. and 34 days p.i., LNs and spleens were harvested and digested in collagenase and DNase as described above. Memory P14 CD8 T cells used as antigen sensors were generated by adoptive transfer of 2×10^3^ naïve Thy1.1^+^ P14 cells into naïve C57BL/6 Thy1.2^+^ recipients that were infected with 5×10^4^ PFU of LCMV the following day. Memory P14 CD8 T cells (>60 days p.i.) were positively enriched by staining splenocytes with phycoerythrin-conjugated anti-Thy1.1 (eBioscience) followed by labeling with anti-phycoerythrin-conjugated magnetic beads (Miltenyi Biotec, Auburn, CA) according to the manufacturer's directions followed by separation via AutoMACS (Miltenyi Biotec). MACS-enriched memory P14 CD8 T cells were CFSE labeled with 10 µM CFSE and co-cultured at either a 1∶10 or a 1∶100 ratio with LN or spleen cells from day 34 LCMV-infected mice for 3 days at 37°C and 5% CO_2_. As a negative control, CFSE-labeled memory P14 CD8 T cells were also co-cultured with either LN or spleen cells from naïve mice. As a positive control for this assay, CFSE-labeled memory P14 CD8 T cells were co-cultured at a 1∶100 ratio with either naïve LN or spleen cells pulsed with 1 µM LCMV GP_33–41_ peptide.

### Real-time PCR

LNs were harvested from naïve, day 4 and day 34 LCMV-infected mice. LNs were homogenized in 1 ml of TRIzol (Invitrogen Life Technologies) and RNA was collected as previously described [36]. Real-time PCR to detect the GP mRNA of LCMV was performed with TaqMan Universal PCR Master Mix (Applied Biosystems) on an ABI 7300 Real Time PCR System (Applied Biosystems) using universal thermal cycling parameters. Results were analyzed using Sequence Detection System Analysis Software (Applied Biosystems). GP gene primers and probe were previously published [27] and purchased from Integrated DNA Technologies. The probe was synthesized to contain FAM reporter dye and 3′-TAMRA quencher dye. Samples were compared with known standard dilutions of a plasmid containing the GP gene of LCMV [40], a gift from Dr. Juan Carlos de la Torre (Scripps Research Institute, San Diego, CA). The number of GP gene copies per LN was calculated based on the number of copies of the GP gene in the sample and the total RNA isolated from the LNs.

### Cell sorting and adoptive transfer of memory CD8 T cells

To purify memory CD62L^+^ and CD62L^−^ P14 CD8 T cells, splenocytes from ≥day 45 LCMV-immune C57BL/6 mice were stained with Thy1.1-PE (Biolegend, San Diego, CA). Cells were subsequently stained with anti-PE magnetic beads (Miltenyi Biotec Inc, Auburn, CA) and positively selected using an AutoMACS (Miltenyi Biotec). AutoMACS-enriched cells were stained for CD8 and CD62L and sorted using a BD FACSAria II (BD Biosciences). Sorted CD62L^+^ P14 CD8 T cells were labeled with 1 µM CFSE (Molecular Probes, Carlsbad, CA) and mixed with unlabeled CD62L^−^ P14 cells at a 1∶1 ratio and 0.75–1.25×10^6^ CFSE-labeled cells were adoptively transferred i.v. into day 34 LCMV immune C57BL/6 (Thy1.2^+^) mice.

## Supporting Information

Figure S1 Cell surface expression of LN homing chemokine receptor CCR7 on memory P14s following an i.p. LCMV infection. Naïve Thy1.1^+^ P14 CD8 T cells were adoptively transferred into naïve Thy1.2^+^ recipients that were subsequently infected i.p. with LCMV 24 h later. The MedLN, ILN, CLN and MesLN were harvested 34 days following infection and transferred cells (CD8^+^Thy1.1^+^) were examined for CCR7 expression. Light gray shaded histograms represent isotype controls. Solid black line histograms represents day 34 LCMV infected mice. Representative data is shown from one of two experiments with four mice per experiment.
(TIFF)

Figure S2 Cell surface expression of β_7_ integrin on memory P14s following an i.p. LCMV infection. Naïve Thy1.1^+^ P14 CD8 T cells were adoptively transferred into naïve Thy1.2^+^ recipients that were subsequently infected i.p. with LCMV 24 h later. The MedLN, ILN, CLN and MesLN were harvested on day 8 or day 34 p.i. and transferred cells (CD8^+^Thy1.1^+^) were examined for β_7_ expression. Dark gray histograms represent day 8 LCMV infected mice. Solid black line histograms represents day 34 LCMV infected mice. Representative data is shown from one of two experiments with four mice per experiment.
(TIFF)

Figure S3 Cell surface expression of activation molecules on memory P14s following an i.p. LCMV infection. Naïve Thy1.1^+^ P14 CD8 T cells were adoptively transferred into naïve Thy1.2^+^ recipients that were subsequently infected i.p. with LCMV 24 h later. The MedLN, ILN, CLN and MesLN were harvested 34 days following infection and transferred cells (CD8^+^Thy1.1^+^) were examined for expression of (A) CD25 and (B) CD69. Light gray shaded histograms represent isotype controls. Solid black line histograms represents day 34 LCMV infected mice. Representative data is shown from one of two experiments with four mice per experiment.
(TIFF)
