# Supplementary figures and images for: The Initial Draining Lymph Node Primes the Bulk of the CD8 T Cell Response and Influences Memory T Cell Trafficking after a Systemic Viral Infection
Source: PLoS Pathog. 2012 Dec 6;8(12):e1003054. doi: 10.1371/journal.ppat.1003054 (PMC3516554; doi:10.1371/journal.ppat.1003054)

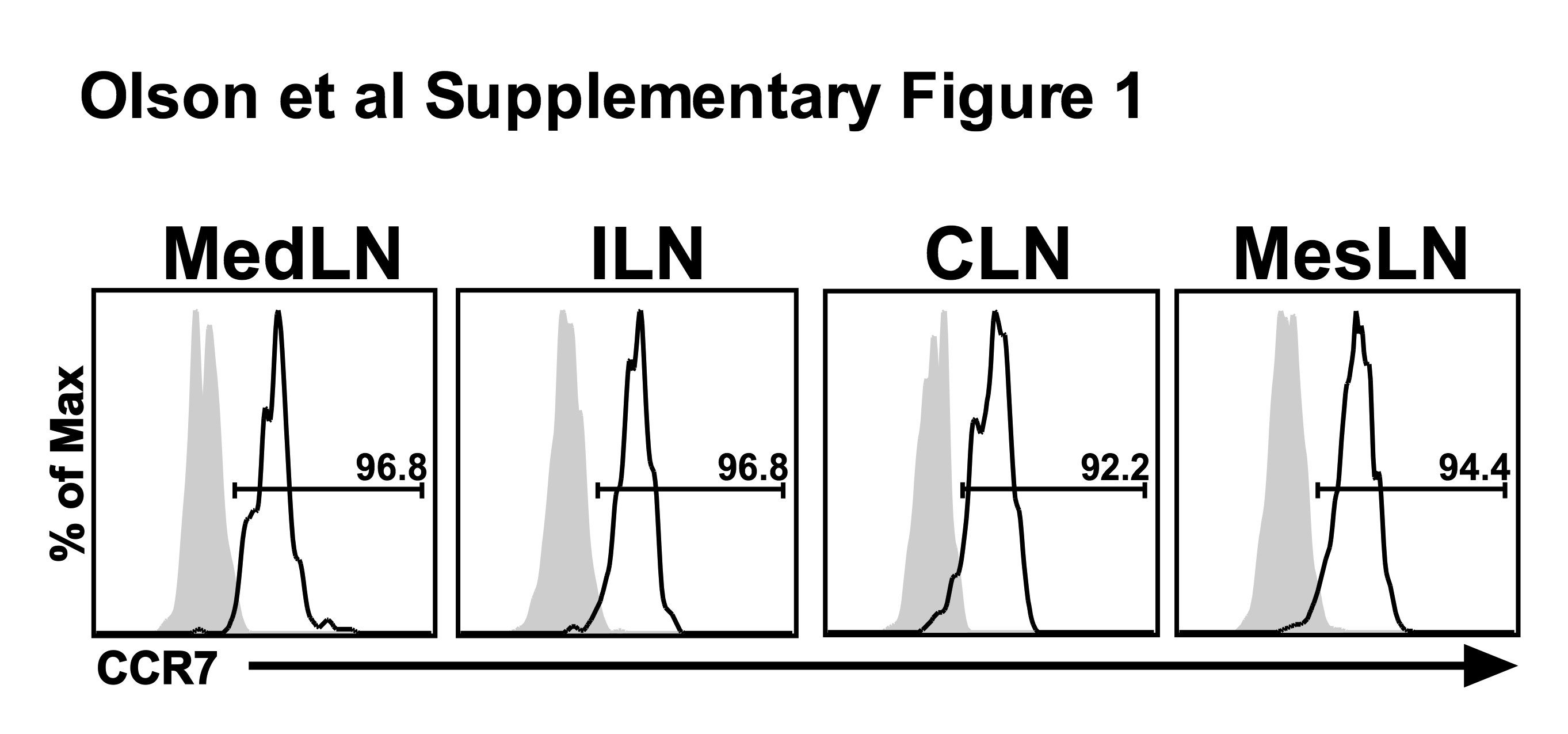

Supplement: Figure S1 — Cell surface expression of LN homing chemokine receptor CCR7 on memory P14s following an i.p. LCMV infection. Naïve Thy1.1+ P14 CD8 T cells were adoptively transferred into naïve Thy1.2+ recipients that were subsequently infected i.p. with LCMV 24 h later. The MedLN, ILN, CLN and MesLN were harvested 34 days following infection and transferred cells (CD8+Thy1.1+) were examined for CCR7 expression. Light gray shaded histograms represent isotype controls. Solid black line histograms represents day 34 LCMV infected mice. Representative data is shown from one of two experiments with four mice per experiment. (TIFF) [file ppat.1003054.s001.tiff]

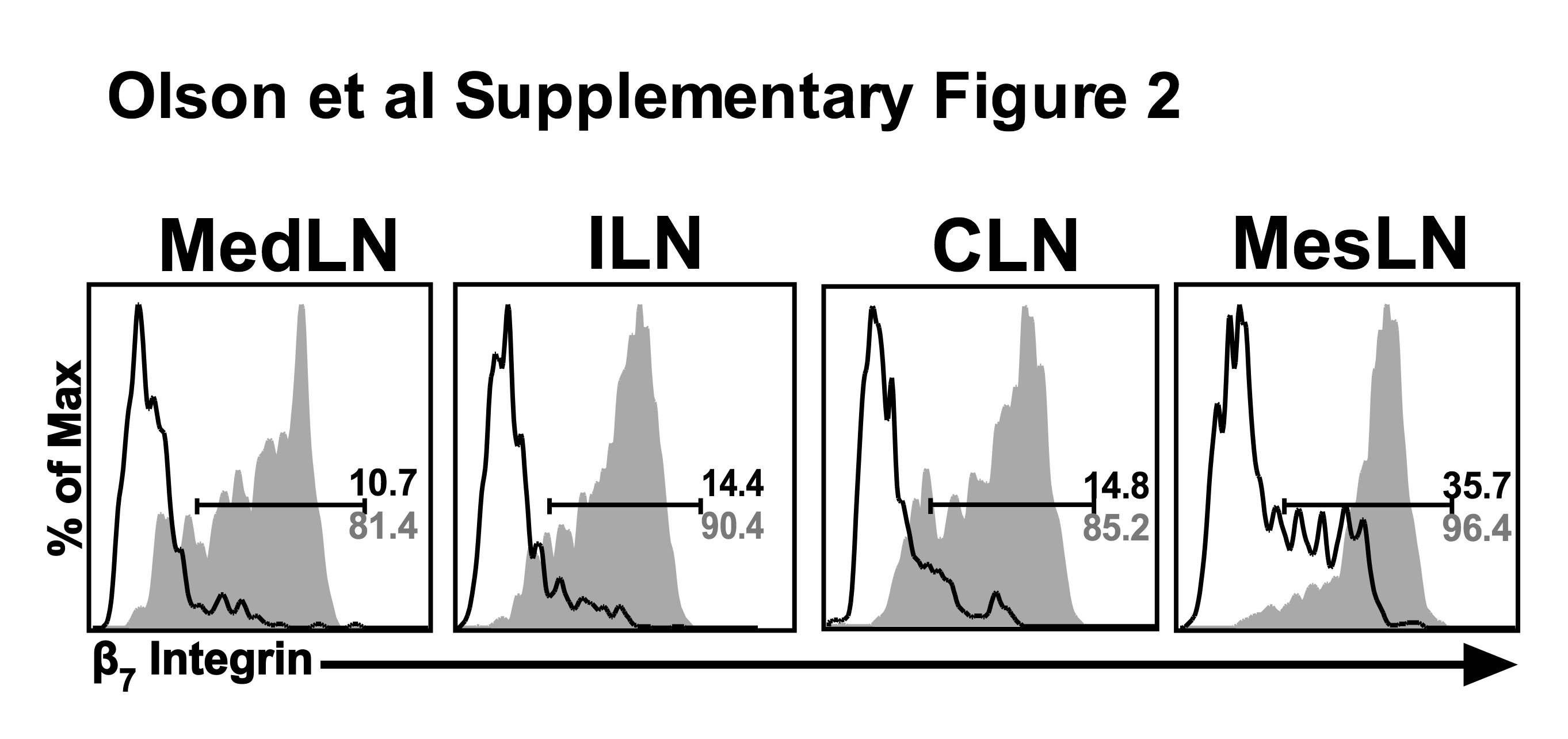

Supplement: Figure S2 — Cell surface expression of β7 integrin on memory P14s following an i.p. LCMV infection. Naïve Thy1.1+ P14 CD8 T cells were adoptively transferred into naïve Thy1.2+ recipients that were subsequently infected i.p. with LCMV 24 h later. The MedLN, ILN, CLN and MesLN were harvested on day 8 or day 34 p.i. and transferred cells (CD8+Thy1.1+) were examined for β7 expression. Dark gray histograms represent day 8 LCMV infected mice. Solid black line histograms represents day 34 LCMV infected mice. Representative data is shown from one of two experiments with four mice per experiment. (TIFF) [file ppat.1003054.s002.tiff]

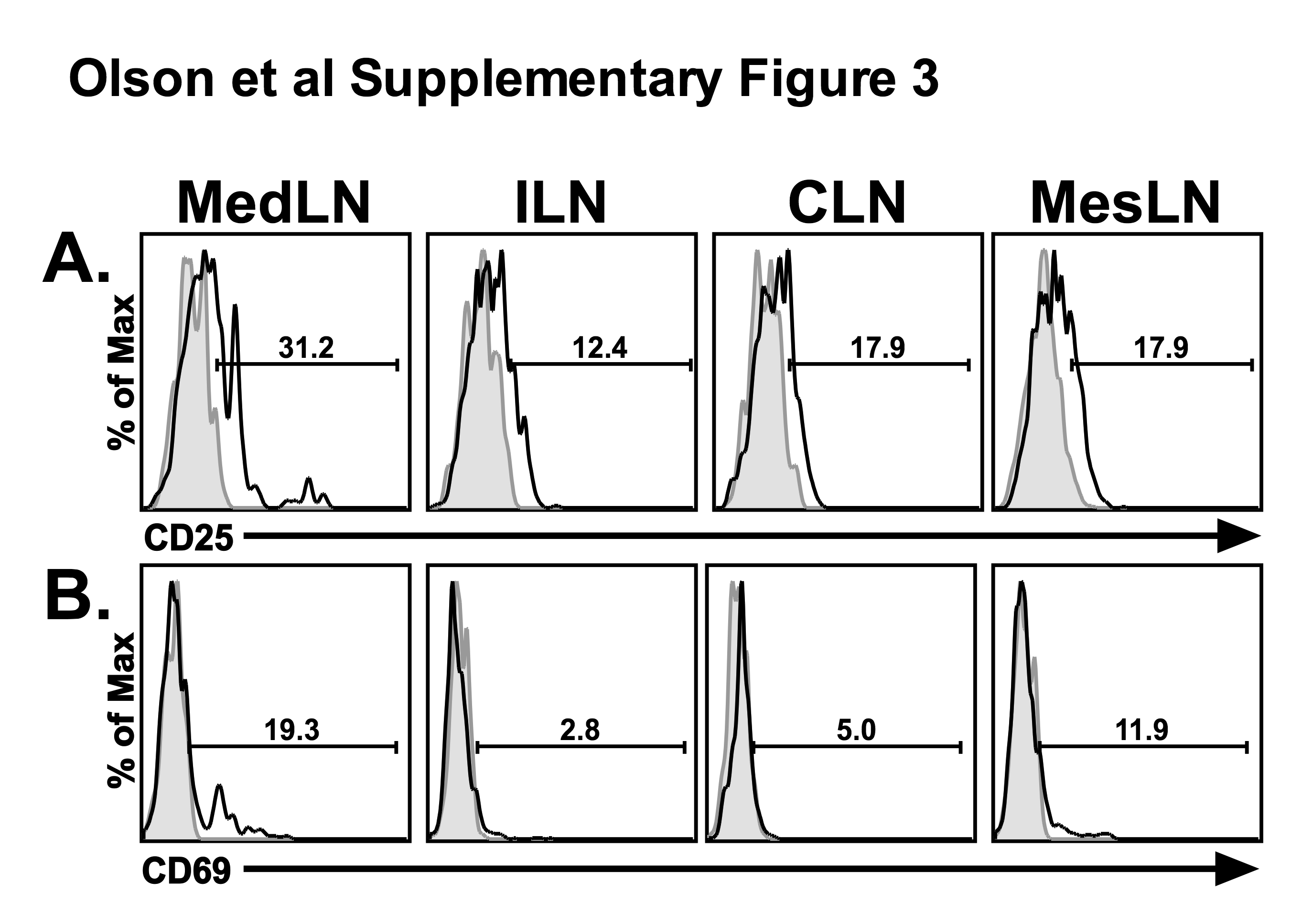

Supplement: Figure S3 — Cell surface expression of activation molecules on memory P14s following an i.p. LCMV infection. Naïve Thy1.1+ P14 CD8 T cells were adoptively transferred into naïve Thy1.2+ recipients that were subsequently infected i.p. with LCMV 24 h later. The MedLN, ILN, CLN and MesLN were harvested 34 days following infection and transferred cells (CD8+Thy1.1+) were examined for expression of (A) CD25 and (B) CD69. Light gray shaded histograms represent isotype controls. Solid black line histograms represents day 34 LCMV infected mice. Representative data is shown from one of two experiments with four mice per experiment. (TIFF) [file ppat.1003054.s003.tiff]
